# Characterization of *Pseudomonas aeruginosa* bacteriophages and control hemorrhagic pneumonia on a mice model

**DOI:** 10.3389/fmicb.2024.1396774

**Published:** 2024-05-14

**Authors:** Yanjie Zhang, Ruiqing Wang, Qingxia Hu, Ni Lv, Likun Zhang, Zengqi Yang, Yefei Zhou, Xinglong Wang

**Affiliations:** ^1^College of Veterinary Medicine, Northwest A&F University, Xianyang, China; ^2^Nanjing Xiao Zhuang University, Nanjing, China

**Keywords:** Pseudomonas aeruginosa, bactericidal activity, complete genome, phage cocktail, hemorrhagic pneumonia

## Abstract

*Pseudomonas aeruginosa* is one of the most common pathogens causing hemorrhagic pneumonia in Chinese forest musk deer. Multidrug-resistant *P. aeruginosa* is frequently isolated from the lungs of affected musk deer in Shaanxi Province, China. With the increasing bacterial drug resistance, commonly used antibiotics have shown limited efficacy against drug-resistant *P. aeruginosa*. Therefore, phages have garnered attention as a promising alternative to antibiotics among researchers. In this study, phages vB_PaeP_YL1 and vB_PaeP_YL2 (respectively referred to as YL1 and YL2) were isolated from mixed sewage samples from a farm. YL1 and YL2 exhibit an icosahedral head and a non-contractile short tail, belonging to the *Podoviridae* family. Identification results demonstrate good tolerance to low temperatures and pH levels, with minimal variation in potency within 30 min of UV irradiation. The MOI for both YL1 and YL2 was 0.1, and their one-step growth curve latent periods were 10 min and 20 min, respectively. Moreover, both single phage and phage cocktail effectively inhibited the growth of the host bacteria *in vitro*, with the phage cocktail showing superior inhibitory effects compared to the single phage. YL1 and YL2 possess double-stranded DNA genomes, with YL1 having a genome size of 72,187 bp and a total G + C content of 55.02%, while YL2 has a genome size of 72,060 bp and a total G + C content of 54.98%. YL1 and YL2 are predicted to have 93 and 92 open reading frames (ORFs), respectively, and no ORFs related to drug resistance or lysogeny were found in both phages. Genome annotation and phylogenetic analysis revealed that YL1 is closely related to vB_PaeP_FBPa1 (ON857943), while YL2 is closely related to vB_PaeP_FBPa1 (ON857943) and Phage26 (NC041907). In a mouse model of hemorrhagic pneumonia, phage cocktail treatment showed better control of the disease and significantly reduced lung bacterial load compared to single phage treatment. Therefore, YL1 and YL2 have the potential for the prevention and treatment of multidrug-resistant *P. aeruginosa* infections.

## 1 Introduction

Forest musk deer was classified as a first-class protected wild animal by Chinese legislation in 2002 and is an important endemic economic animal. As a small ruminant, the forest musk deer often experiences accidental injuries, which frequently lead to the occurrence of suppurative infections in the skin, liver, and lungs (Guan et al., 2009; Yang et al., 2023). Research has shown that suppurative infections are one of the main obstacles to forest musk deer reproduction (Lu et al., 2023). According to a previous report on forest musk deer treatment, a farm in Sichuan Province, China, had 35.3% (165/467) cases related to suppurative infections out of forest musk deer treatment records. Furthermore, 40% (40/100) death cases were diagnosed as severe internal suppuration, with the lungs being the main site of infection. Suppurative infections in the skin of forest musk deer are easily observed, but suppurative infections in internal organs are often difficult to detect in a timely manner, leading to delays in treatment and missing the optimal treatment opportunities (Zhao et al., 2011; Zhao K. L. et al., 2019).

*Pseudomonas aeruginosa* is a gram-negative opportunistic pathogen that is widely present in the environment. With the emergence of multidrug-resistant *P. aeruginosa*, the effectiveness of antibiotic inhibition has become weaker (Qi et al., 2014). *P. aeruginosa*’s resistance is enhanced by the presence of broad-spectrum β-lactamases (ESBL) in its genome, which inactivate β-lactam antibiotics (Moya et al., 2009). Additionally, multidrug efflux pump systems and difficult-to-remove biofilms are also considered major factors contributing to *P. aeruginosa*’s resistance (Ziha-Zarifi et al., 1999). Although there are currently multivalent vaccines available for *P. aeruginosa*-induced hemorrhagic pneumonia, they are still limited by their short duration of protection (Hammer et al., 2003). Therefore, it is necessary to develop new antibacterial drugs to control *P. aeruginosa* infections considering the aforementioned reasons. Bacteriophages, as the most abundant viruses in nature (Cao et al., 2015), have a broad host range and can kill various host bacteria (Gong et al., 2017). Currently, bacteriophages have been gradually considered as alternatives to antibiotic treatment for *P. aeruginosa* infections due to their targeted host specificity and rapid proliferation advantages (Karumidze et al., 2012). With the emergence of multidrug-resistant bacteria, phage cocktails composed of multiple phage strains have become a preferred method to improve the efficiency and effectiveness of antibacterial treatment (Petsong et al., 2019). Some research groups have reported the feasibility of phage therapy for *P. aeruginosa* infections in animals (Shi et al., 2020; Yin et al., 2022; Cui et al., 2023). However, according to our literature review, Cao et al. (2015) and Gu et al. (2016) extensively investigated and evaluated the therapeutic efficacy of nebulized or nasal-drip bacteriophages in a mink model of hemorrhagic pneumonia caused by *P. aeruginosa*.

In this study, we isolated pathogenic *P. aeruginosa* from the lung of a forest musk deer with hemorrhagic pneumonia, and used it as the host bacterium to isolate two lytic *P. aeruginosa* bacteriophages. We characterized the morphological and biological features of phages YL1 and YL2, and also applied them in a mice model of hemorrhagic pneumonia, aiming to provide a basis for the development of bacteriophage therapy for multidrug-resistant *P. aeruginosa* infections in wild economic animals.

## 2 Materials and methods

### 2.1 Animals

SPF Balb/c mice at 6 weeks of age were purchased from Chengdu Chengda Shuo Experimental Animal Co., Ltd. in Sichuan, China. All animal experiments in this study complied with the guidelines of the Chinese Animal Welfare Committee and were approved by the Animal Welfare and Research Ethics Committee of Northwest A&F University in Shaanxi, China.

### 2.2 Bacterial strains and growth conditions

The *P. aeruginosa* strains used in this study were previously isolated and kept in the Epidemic Prevention and Control Laboratory of Northwest Agriculture and Forestry University. All 165 strains were previously cultured by cetyltrimethylamine bromide (Qingdao Haibo Biological Company, China) and PCR. Other isolates were isolated, identified and preserved by the Epidemic Prevention and Control Laboratory of Northwest A&F University. All strains were cultured in LB broth at 37°C with 50% glycerol in sterile LB broth and stored in a −80°C refrigerator.

### 2.3 Isolation and purification of phages

The isolation and purification of the phage was performed using a bilayer plate method (Wang et al., 2017). Manure, soil and sewage were collected from a forest musk farm in Shaanxi Province, China. Manure and soil were soaked in saline overnight and initially filtered through three layers of gauze, and the supernatant was retained. The effluent samples were centrifuged at 6,000 rpm for 10 min and the supernatant was taken. The two supernatants were mixed with 300 μL of indicator bacteria of *P. aeruginosa* 27 (PA27) in 100 mL LB broth, incubated overnight at 37°C, and then centrifuged at 12,000 rpm for 10 min. The supernatant was collected and filtered through a 0.22 μm membrane to obtain the phage enrichment solution. One hundred microliters of the stock solution was mixed with the same volume of PA27 bacterial solution and incubated at 37°C for 5 min. Finally, 200 μL of the mixture was mixed with 5 mL of melted LB agar at 50°C and poured on top of the LB plates. The plates were left to stand for 10 min and placed to incubate at 37°C for 12 h to produce plaques. Individual phage plaques were selected and dissolved in 100 μL of SM buffer for 30 min at 37°C, then centrifuged at 12,000 rpm for 10 min. The supernatant was taken for 10-fold ratio dilution and purified at least 5 times by double layer plate method to obtain phage plaques of uniform size and shape.

### 2.4 TEM observation of phage morphology

The morphology of phages YL1 and YL2 was determined by transmission electron microscopy (TEM) observation (Ackermann, 2009). Specifically, 20 μL of phage suspension (3.3 × 10^11^ PFU/mL) was dropped on the surface of a copper mesh and adsorbed for 15 min. Then, the phage was stained with 2% phosphotungstic acid for 5 min, washed three times with ddH_2_O, and dried before observing the phage and recording the images using a HT7700 transmission electron microscope (TEM, Hitachi, Japan).

### 2.5 Host range determination

The host range of phage YL1 and YL2 was tested using a double-layer agar plate method with 165 strains of *P. aeruginosa* and other strains. The tested bacterial broth was freshly prepared at 10^8^ CFU/mL. A 100 μL volume of phage YL1 and YL2 was taken and mixed with the same volume of the tested bacterial broth, and then incubated at 37°C for 5 min. Finally, the phage titers were measured as above.

### 2.6 One-step growth curve

One-step growth curves were experimented as previously reported, with some modifications (Lee et al., 2021). Phages YL1 and YL2 were mixed with the host strain of PA27 at optimal infection rate (MOI) respectively and then incubated at 37°C for 5 min. The mixture was centrifuged at 12,000 rpm for 30 s and washed twice with sterile LB broth to remove unabsorbed phages. The sediment was resuspended in 5 mL of LB broth and incubated at 37°C with shaking at 200 r/min. After inoculation with phage, samples were taken every 5 min for the first 30 min and every 10 min for the next 170 min. The titer of phage was measured after centrifugation at 12,000 rpm for 5 min.

### 2.7 Determination of phage temperature, pH and UV stability

To investigate the effect of different relevant environmental conditions on phage infection, phage stability at various conditions should also be assessed. We incubated aliquots of the two phage proliferators with determined titers in a water bath at a range of temperatures (−20°C, 4°C, 30°C, 40°C, 50°C, 60°C, and 70°C) for 1 h. Three parallel controls were set up at each temperature and titers were measured using the double layer agar plate method. In addition, two strains of phage were exposed to sterile petri dishes, which were then placed in an ultra-clean bench for 2 h of UV irradiation, and samples were taken at 10 min intervals during the first 1 h and at 20 min intervals during the second 1 h and titers were measured using the double-layer agar plate method to determine the stability of the phage. Prepare 1 mol/L of NaOH solution and HCl solution and use a burette to configure sterile LB broth with different pH values of 2, 4, 6, 8, 10 and 12, add 1 mL of phage proliferation solution of known potency to it, adjust its pH to 7 using NaOH solution and HCl solution immediately after 1 h of water bath at 37°C and measure the titer using the double-layer agar plate method. The experiment was repeated three times.

### 2.8 Phage genome bioinformatics analysis

Genomic DNA of the two phage strains was extracted by phenol-chloroform method (Zhao F. et al., 2019). We analyzed phage genome sequences using NCBI ORF finder and GeneMarks for prediction and annotation of phage open reading frames (ORFs) (Besemer et al., 2001). The predicted ORFs were identified by comparison with BLASTp in NCBI. Phage tRNAs were predicted using tRNAscan-s (Schattner et al., 2005). Lysogenic genes in the genome were analyzed using the online tool PHASTER. The online tool PhageScope server allows analysis of phage drug resistance genes and virulence genes, and the whole genome of phage was mapped in circles using CGview online software. The phylogenetic tree of the two phage strains was constructed using MEGA 10 (Tamura et al., 2013) based on conserved and meaningful sequences.

### 2.9 *In vitro* bactericidal activity

To understand the bactericidal activity of the two strains of *P. aeruginosa in vitro*, PA27 was used as the host bacterium and incubated at 37°C until the logarithmic growth phase of the bacteria, and the concentration of the bacteriophage solution was measured, and the potency of the phage proliferation solution was also determined; the proliferation solution was centrifuged at 8,000 rpm for 5 min, the supernatant was discarded, and new sterile LB broth was added and resuspended three times. In a 96-well bacterial culture plate, 50 μL of the above resuspension solution was added to each well, and then 50 μL of single phage or phage cocktail was added to each well, and the test was performed according to phage OMOI, and the phage cocktail was mixed in a 1:1 volume ratio, and three parallel tests were set up for each group. The control group was spiked with 100 μL of bacterial solution and 100 μL of sterile LB broth and incubated in a 37°C incubator, and OD_600_ was measured every 1 h. The experiment was performed three times. All data were statistically analyzed using GraphPad Prism 8.0 software.

### 2.10 Animal study

To assess the therapeutic ability of two strains of phages against infection with *P. aeruginosa* haemorrhagic pneumonia, a mouse model of haemorrhagic pneumonia was constructed and given treatment. To determine the half lethal dose (MLD_50_) of the host bacterium PA27, five Balb/c mice in each group were intranasally challenged with 50 μL PA27 (3.5 × 10^9^, 3 × 10^8^, 3.5 × 10^7^, 3 × 10^6^ or 3.5 × 10^5^ CFU/mL) in a nasal drip after respiratory anesthesia via isoflurane. As a control, another group of mice was treated with PBS. The number of dead mice was recorded daily during the 7-day follow up period. All of the assays were performed in triplicate. According to the measured median lethal dose in mice, the experimental group was inoculated with 50 μL of *P. aeruginosa* PA27 (10^8^ CFU/mL). After a 2 h challenge with PA27, mice were respiratory anesthetized with isoflurane and injected intranasally with either YL1 (10^8^ PFU/mL in a volume of 50 μL), YL2 (10^8^ PFU/mL in a volume of 50 μL) or a cocktail (YL1 and YL2,10^8^ PFU/mL in a volume of 50 μL). Each dose group consisted of five Balb/c mice. As a control, one group of infected Balb/c mice was treated with 1 mL of PBS. Each group was treated for 24 h, after which euthanasia was performed. All assays were performed in triplicate.

To monitor the amount of bacteria in the lungs of mice, lung tissue samples were also collected from euthanized mice. The lungs were weighed, suspended in 1 mL of aseptic PBS, and homogenized using a tissue grinder. Viable PA27 cell counts (CFU/g tissue) were determined by serial dilution and coating on LB agar plates. Histopathological analysis was performed on the lungs of PA27-infected mice treated with phage or PBS and on the lungs of uninfected mice. Mice were euthanized 24 h after PA27 challenge. Lung tissues were removed and immediately placed in 4% paraformaldehyde. Paraformaldehyde-fixed tissues were processed and analyzed by microscopy.

## 3 Results

### 3.1 Morphology and host range

**Figure 1:**
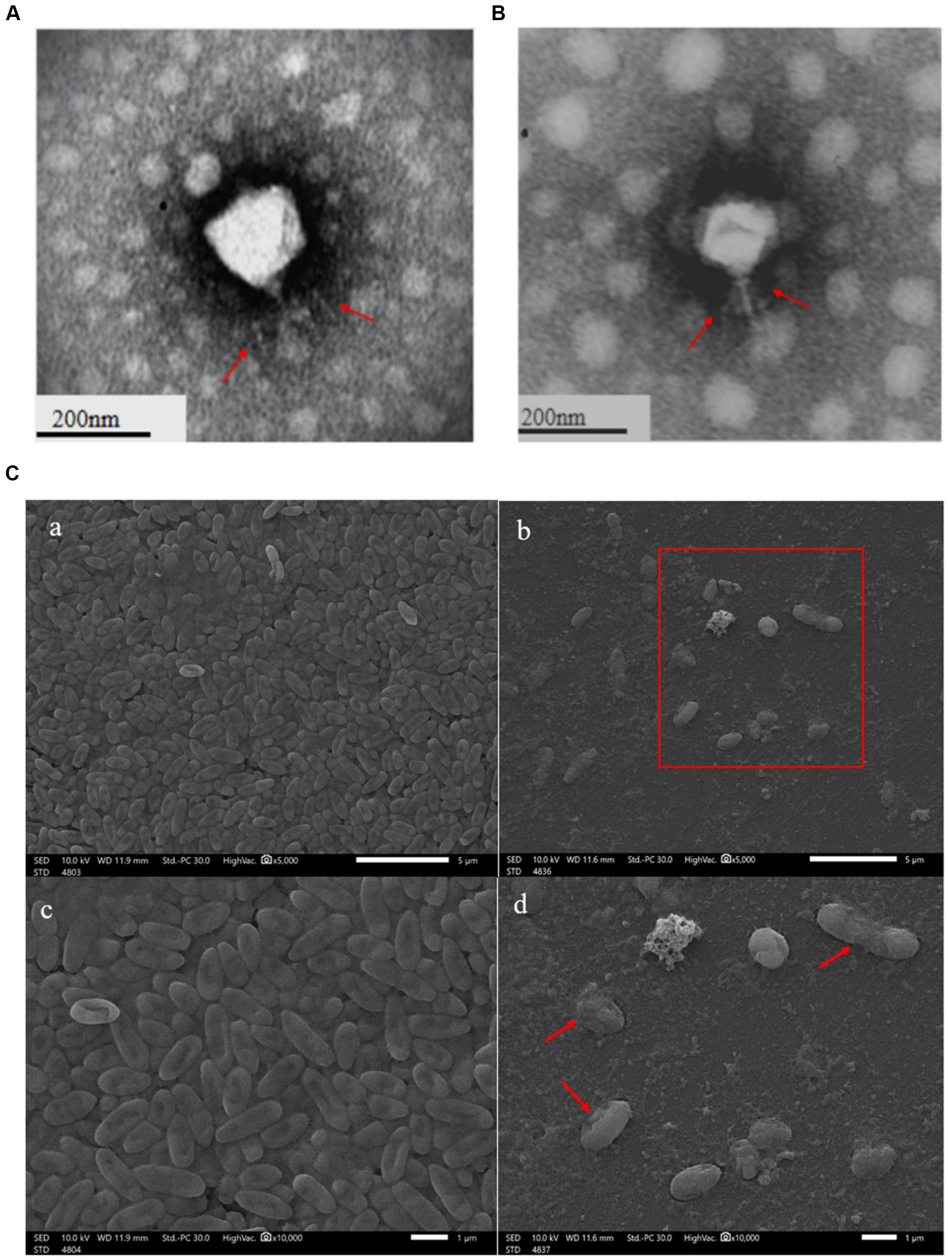
Transmission electron microscopy of phage YL1 **(A)** and YL2 **(B)**. SEM image of bacteriophage lysing *Pseudomonas aeruginosa* **(C)**.

The clear plaques produced by the phage YL1or YL2 that infected PA27 strain had well-defined boundaries with a diameter of about 3 mm (Supplementary datasheet 1). TEM images showed that the phage YL1 possessed an isometric polyhedral head of about 70.3 nm in diameter and an uncontracted short tail of approximately 33 nm, and the phage YL2 possessed an same head like YL1 about 65 nm in diameter and an uncontracted short tail of approximately 35 nm (Figures 1A,B). According to the current classification system developed by the International Committee on the Taxonomy of Viruses (ICTV), they belong to *Podoviridae* family and designated as vB_PaeP_YL1 and vB_PaeP_YL2 (abbreviated of YL1 and YL2). The state of *P. aeruginosa* PA27 after cracking by phage can be lysed seen under SEM (Figure 1C).

YL1 and YL2 exhibited lytic activity. The results showed that the lytic rate of phage YL1 to different sources of *P. aeruginosa* strains was 40.6% (67/165), while that of phage YL2 to different sources of *P. aeruginosa* strains was 32.1% (53/165) (Supplementary datasheet 2, PA12 is the standard *P. aeruginosa* strain). There was no cross-species cleavage in the two bacteriophages. To broaden the bactericidal spectrum, a bacteriophage cocktail was constructed by considering the optimal MOI of the two phages, as well as the principle of host range complementarity among bacteriophages.

### 3.2 Characterization of phage YL1 and YL2

**Figure 2:**
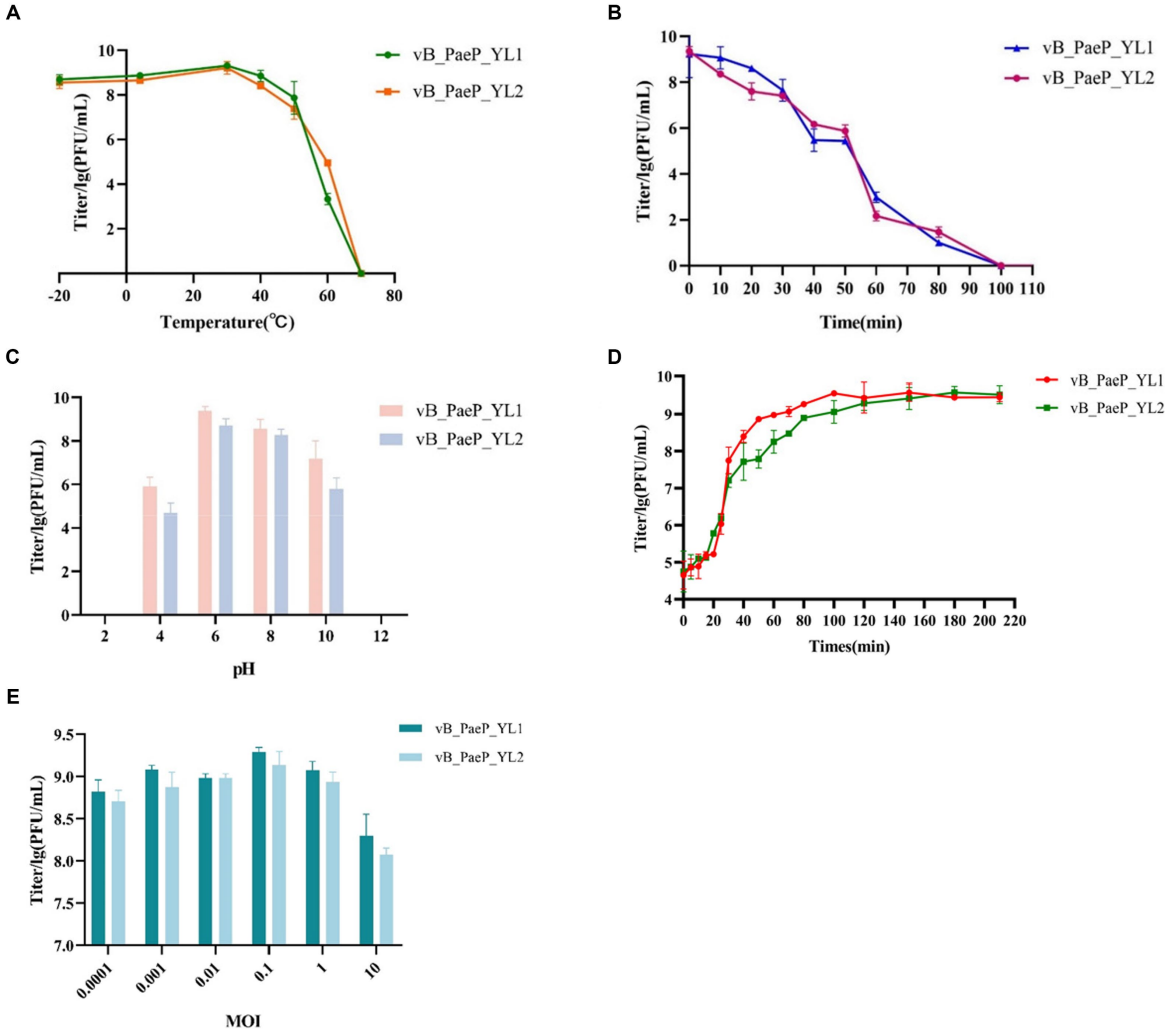
Biological characteristics of phage YL1 and YL2. **(A)** Temperature sensitivity of YL1 and YL2. **(B)** UV stability of phage YL1 and YL2. **(C)** pH stability of YL1 and YL2. **(D)** One-step growth curve of YL1 and YL2. **(E)** Determining the optimal multiplicity of infection of phage. Data are expressed as mean ± SD (*n* = 3).

The thermal stability test showed that YL1 was stable below 50°C, but it was completely inactivated at 70°C after 1 h (Figure 2A). In addition, the titer of YL1 could be maintained above 10^6^ PFU/mL at 60°C. The survival rate of YL2 is same like YL1. The stability of YL1 and YL2 under ultraviolet light was studied. The results showed that the titers of YL1 and YL2 had been in a downward trend until 120 min, but the titers of the two bacteriophages dropped to 0, suggesting that they were sensitive to UV irradiation (Figure 2B). YL1 and YL2 could maintain activity over a broad range of pHs (4–10) (Figure 2C), but it was inactivated at extreme pHs (below pH 4 or above pH 10), indicating that YL1 and YL2 has a good acid–base tolerance. Additionally, the one-step growth curve of YL1 revealed that the latent period and lysis period were 10 and 90 min, respectively, and the average burst size was 125 PFU/infected cell. The latent period and lysis period of YL2 were 15 and 80 min, and the average burst size was 94 PFU/infected cell (Figure 2D). The MOI for both YL1 and YL2 was 0.1 (Figure 2E).

### 3.3 *In vitro* bactericidal activity

**Figure 3:**
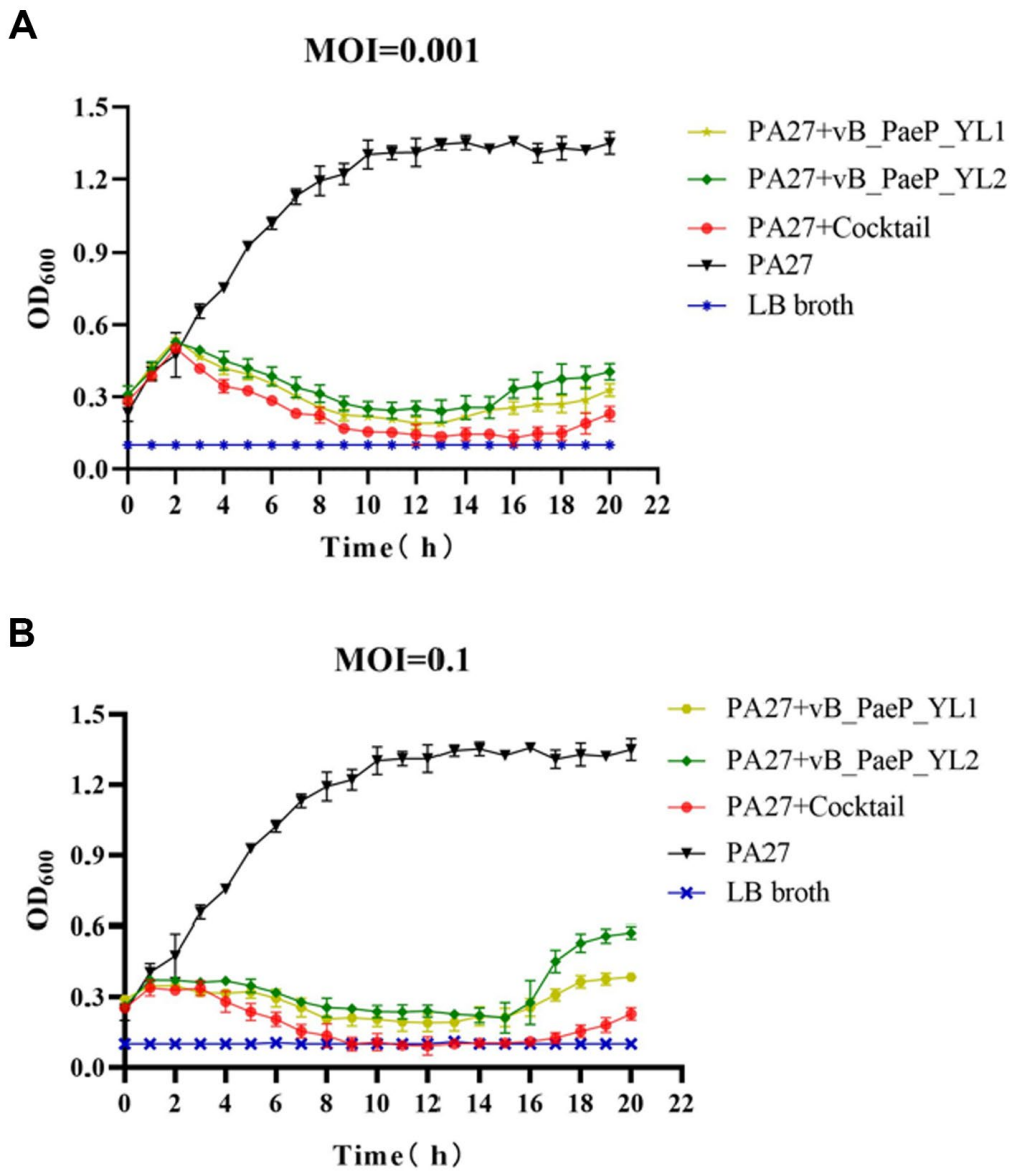
Bactericidal activity of bacteriophage YL1 and YL2 against PA27 *in vitro* under different infection conditions (MOI = 0.001 or 0.1). The data are expressed as means ± SD (*n* = 3).

As shown by the trend of the curve (Figures 3A,B), the OD_600_ values of the positive control increased continuously within 24 h, and the OD_600_ values of the negative control remained unchanged, the OD_600_ values of the phage cocktail group continued to rise within 1–3 h regardless of whether the MOI was 0.1 or 0.001. Between 3–9 h, the OD_600_ values of each individual phage group began to decline, with the phage cocktail group showing a more significant decline. Between 9–15 h, the OD_600_ values of each phage group tended to stabilize. Between 15–20 h, the OD_600_ values of the YL1 and YL2 groups slowly increased, suggesting that this may be due to a buildup of bacterial fragments in the sample or the emergence of phage-resistant strains, leading to an increase in OD_600_ values. In short, both single phages and the phage cocktail group effectively inhibited the growth of the host bacteria PA27 *in vitro*, with the phage cocktail group exhibiting a superior inhibitory effect compared to the single phage groups.

### 3.4 Complete genome analysis of phage

#### 3.4.1 Genomic characteristics of phage

Compared to the genomes in the NCBI database, the phage YL1 showed 97.24% similarity to *P. aeruginosa* phage Pa2, while bacteriophage YL2 showed 97.09% similarity to *P. aeruginosa* phage vB_PaeP_TUMS_P121. Both phages are double-stranded DNA phages. The genome size of YL1 was 72,187 bp with a total G + C content of 55.02%, while the genome size of YL2 was 72,060 bp with a total G + C content of 54.98%. YL1 and YL2 predicted 93 and 92 meaningful putative open reading frames (ORFs), respectively, and no ORFs related to drug resistance or lysis were found in either phage genome (Supplementary datasheet 3). Among them, 82 ORFs (88.17%) in phage YL1 contained an ATG start codon, 8 ORFs contained a GTG start codon, and 3 ORFs contained a TTG start codon, whereas phage YL2 contained 83 ORFs (90.21%) with an ATG start codon, 8 ORFs with a GTG start codon, and 1 ORF with a TTG start codon. The complete genome sequences of phages YL1 and YL2 have been respectively deposited in GenBank under accession numbers OQ992204 and OQ992205.

#### 3.4.2 Functions of ORFs

**Figure 4:**
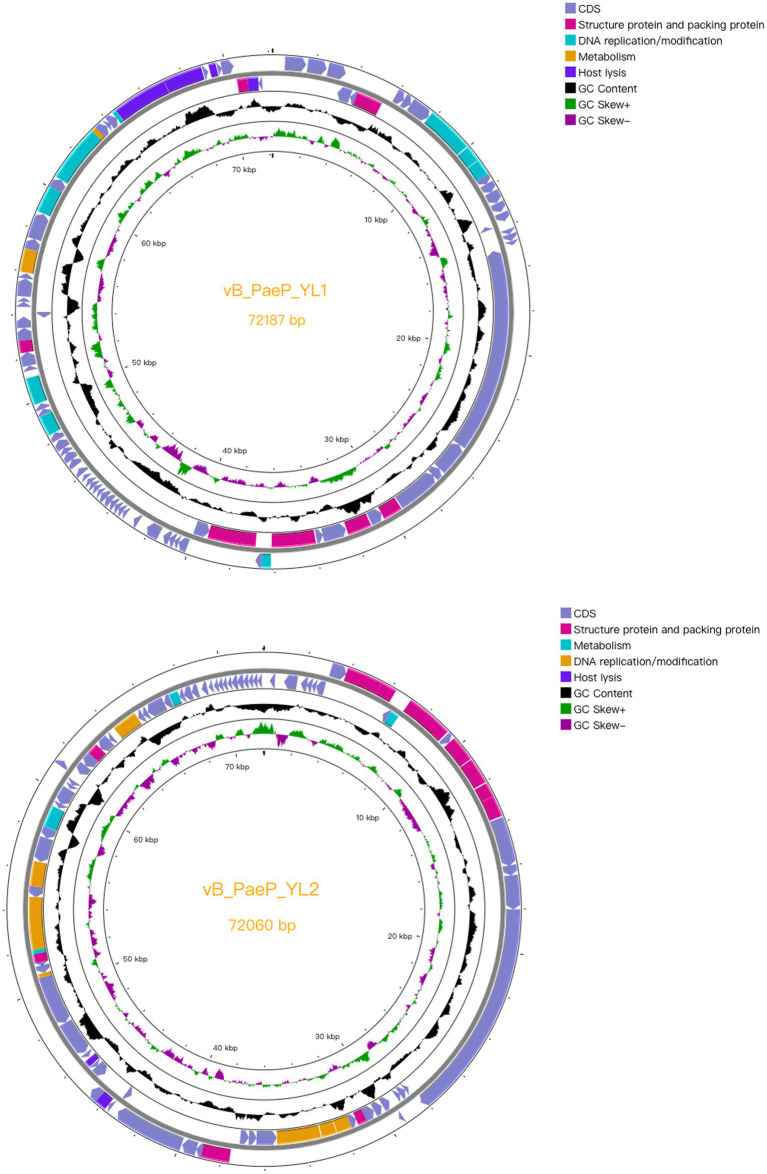
Complete genome structure of phage YL1 and YL2. The outermost circle is the ORF encoded by the phage YL1 or YL2. Arrows represent gene transcription directions. The pink areas represent structural and packaging proteins, the blue areas represent host lytic genes, the cyan regions represent DNA duplicating or modifying genes and the orange areas represent metabolic genes.

Both phages are double-stranded DNA phages and have a modular genome structure similar to that of double-stranded DNA phages, as shown in Figure 4, including structural and packaging proteins, host lysis genes, DNA replication/modification genes, and metabolic genes. Details are listed in Supplementary datasheet 3. The structural proteins, including tail fiber protein, capsid and scaffold protein, major capsid protein, portal protein, and terminase large subunits. Generally, the terminase consists of two subunits (large and small subunits) that are involved in DNA packaging (Catalano, 2000). In the YL1 and YL2 genomes, the same large subunit of terminase is encoded by ORF35 and ORF8, respectively, and they share high similarity with the large subunit of terminase in vB_PaeP_TUMS_P121. ORF29 in YL1 and ORF14 in YL2 encode tail length tape measure proteins, which play a role in contacting the inner membrane and periplasmic proteins when injecting the phage genome into bacteria during infection. They determine the length of the phage tail and facilitate DNA transfer into the cytoplasm during infection. The fact that both phages encode the same tail fiber protein suggests that they can recognize similar host bacteria species and may have similar biological characteristics.

The DNA replication and modification proteins of two phages, such as DNA helicase, DNA primase, DNA polymerase, and HNH endonuclease, are identical and play a critical role in DNA double strand cleavage, DNA replication fork formation, DNA repair, and transcription. The HNH endonucleases encoded by YL1 (ORF84) and YL2 (ORF51) belong to the phage DNA replication and modification-related proteins. It was identified and reported that HNH endonucleases (gp74) are required for the specific endonuclease activity of the phage HK97 terminase and are essential for the head morphogenesis of the phage (Kala et al., 2014). Bioinformatic analysis of Kala et al. (2014) revealed that the role of HNH proteins in terminase function is widespread among long-tailed phages and is uniquely required for the activity of the Terminase1 family of large terminase proteins.

Holin accumulates in the cell membrane and forms holes to activate endolysin to enter the cell wall. After reaching the cell wall, endolysin hydrolyzes peptidoglycan, and spanin is activated to undergo a conformational change and destroy the outer membrane. Phage YL1 encodes Rz-like spanin protein, which has a transmembrane domain at the N terminus located on the inner membrane. The Rz1 encoding region can be independent, embedded or overlapped with Rz, and these two proteins can form a complex, which is the last step required for host lysis (Summer et al., 2007). ORF54 of bacteriophage YL2 is the cytidine and deoxycytidylate deaminase zinc-binding region. Cytidine and deoxycytidine deaminase (CDA) is an important enzyme that plays a critical role in maintaining the balance of DNA and RNA base composition. The zinc-binding region in CDA enzymes refers to a unique structural region in the molecule that is involved in the catalytic reaction with the zinc ion. This region consists of multiple conserved amino acids that are highly conserved across different members of the CDA enzyme family (Pace and Weerapana, 2014).

Since the lysis systems of the two phages are the same, we only analyse the holin and lysozyme encoded by ORF88 and ORF92, respectively, in phage YL1. Transmembrane prediction of YL1 holin (Supplementary datasheet 4). shows that it is a transmembrane protein with hydrophobic transmembrane domains (TMDs) and has three transmembrane regions. The N terminus of the protein extends to the periplasmic space, and the C terminus is located in the cytoplasm. According to its characteristics, we classify it as a type I perforin protein. According to the prediction, the YL1 lysin does not have a TMD or signal peptide. Like most phage lysins, it cannot directly penetrate the host cell membrane. It could rely on the auxiliary role of phage-encoded pore-forming proteins to pass through the host cell membrane and act on the peptidoglycan layer of the cell wall, hydrolyzing and destroying the cell wall to play its role in lysing bacteria.

#### 3.4.3 Phylogenetic analysis

**Figure 5:**
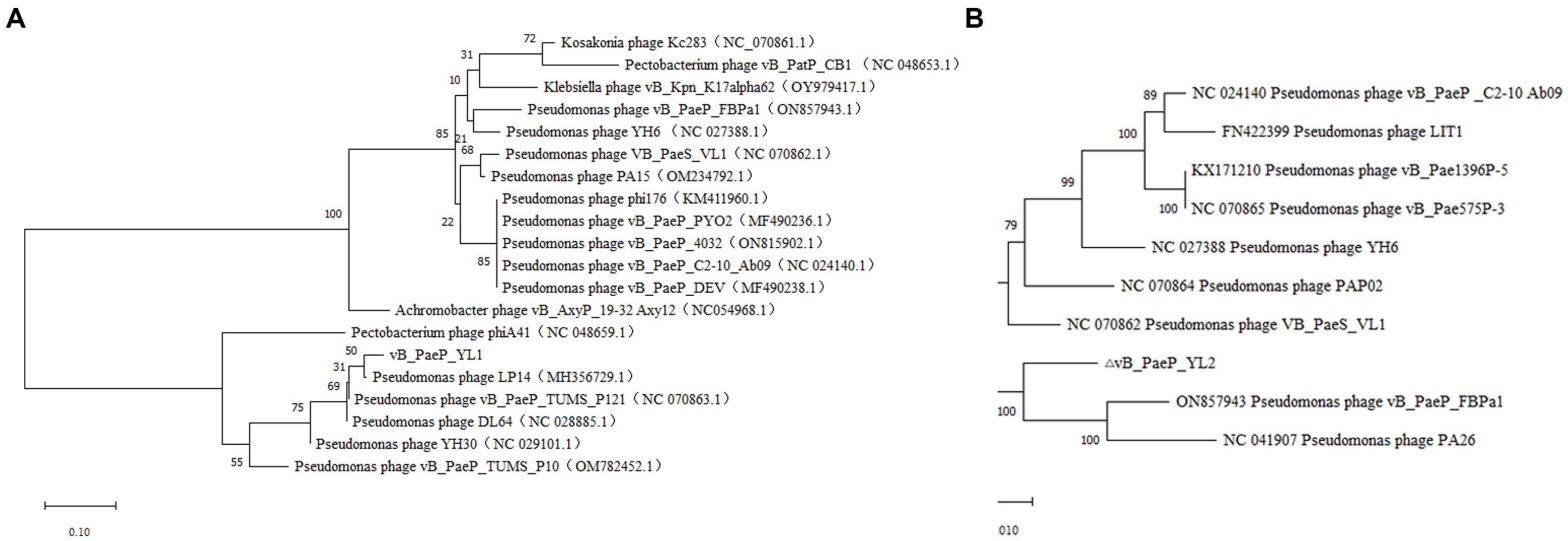
Phylogenetic analysis of phage YL1 and YL2. Phylogenetic tree based on the large subunit terminal enzymes of vB_PaeP_YL1 **(A)**, and the helicase of vB_PaeP_YL2 **(B)** were compared using MEGA11.

BLASTp analysis showed that 25 ORFs in phages YL1 and YL2 were annotated as encoding proteins with known functions. To investigate the evolutionary relationship between the two phages and other phages, we constructed a phylogenetic tree of the large subunit terminal enzymes of YL1 and DNA helicase of YL2, respectively. As shown in Figure 5, large subunit terminal enzymes of YL1 was most closely related to LP14 (MH356729), while DNA helicase of YL2 was most closely related to vB_PaeP_FBPa1 (ON857943) and Phage26 (NC041907). Therefore, based on sequence, phylogenetic relationship, genome size and structure, as well as the ICTV virus classification system, both phages belong to the *Litunavirus* genus (Turner et al., 2021), which confirms their close evolutionary relationship.

### 3.5 Phage protection in mice with hemorrhagic pneumonia

**Figure 6:**
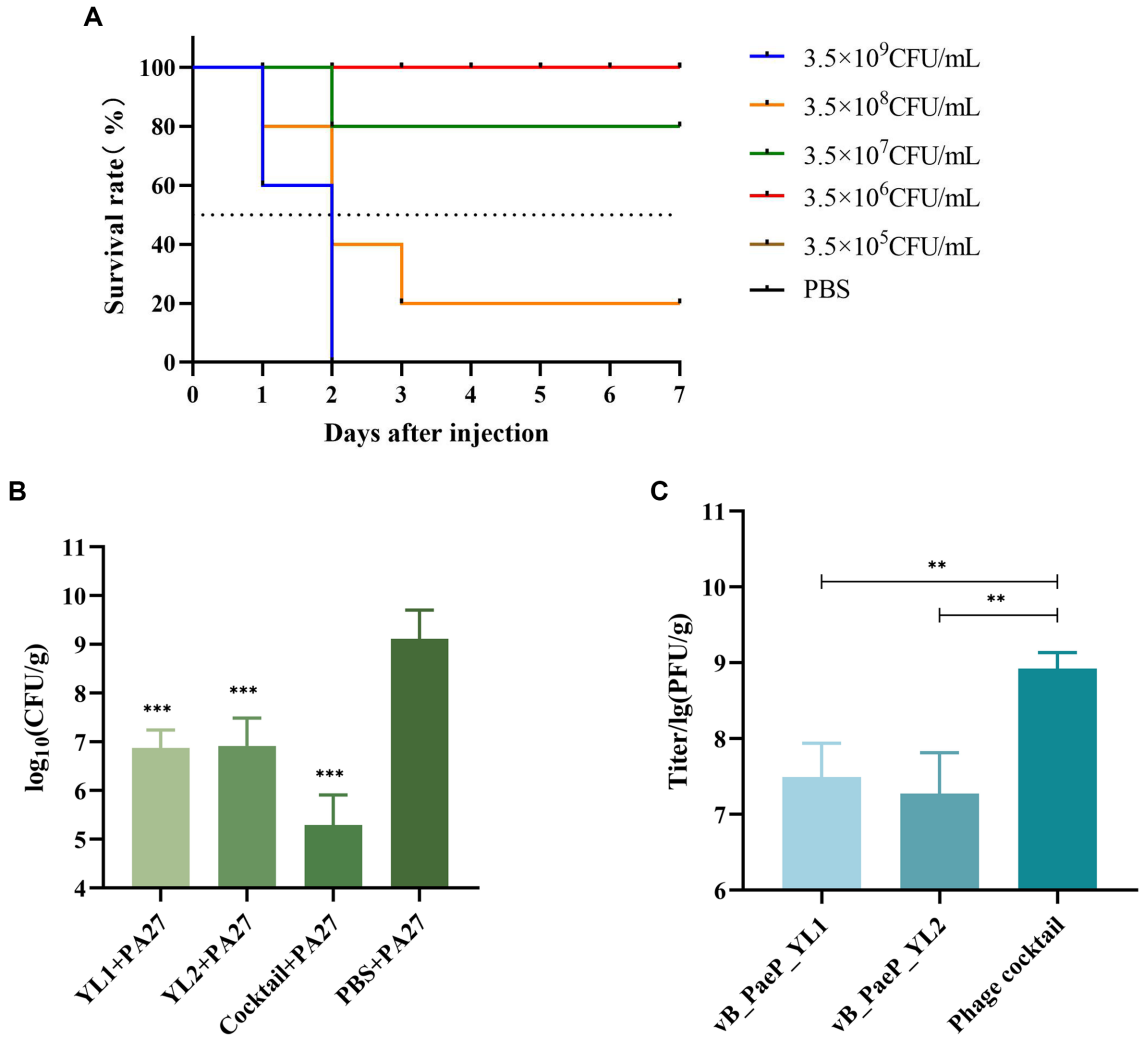
The indicator strain PA27 mouse MLD_50_ was determined **(A)**. Two hours after infection, mice were treated with phage YL1, YL2, cocktail or PBS, and healthy lungs were used as control group. The changes of lung bacterial load **(B)** and phage load **(C)** were measured 24 h later (^**^p < 0.05, ^***^p < 0.001).

Each mouse was injected with 3.5 × 10^9^ CFU/mL of PA27, which was sufficient to induce hemorrhagic pneumonia and resulted in 100% mortality within two days. However, mice injected with 3.5 × 10^7^ CFU/mL of *P. aeruginosa* PA27 showed a survival rate of 80%. Based on the Reed–Muench formula, the mouse median lethal dose (MLD_50_) was determined to be 1.5 × 10^8^ CFU/mL (Figure 6A). To determine the protective effect of phage and phage cocktail against hemorrhagic pneumonia in mice, mice were intranasally administered with phage YL1 (10^8^ PFU/mL), phage YL2 (10^8^ PFU/mL), and phage cocktail (10^8^ PFU/mL) 2 h after challenge with *P. aeruginosa*. Compared to PBS, phage significantly protected mice from *P. aeruginosa* infection. After 24 h of treatment, the mice were euthanized and the bacterial counts in their lungs were measured. In comparison to the PBS group, the single phage-treated mice exhibited a reduction in bacterial load by 2 to 3 log values, while the phage cocktail-treated group showed a decrease by 4 log values. Notably, no bacterial colonies were detected in the lungs of mice from the normal control group. It was found that the administration of phage significantly reduced the bacterial load in the lungs, with the phage cocktail group showing the most significant reduction (Figure 6B). Phage load assay revealed a slight decrease in the number of individual phage in the lungs, while the phage cocktail showed an increase, confirming the effective infection of host strain PA27 by both individual phage and phage cocktail in mice (Figure 6C).

**Figure 7:**
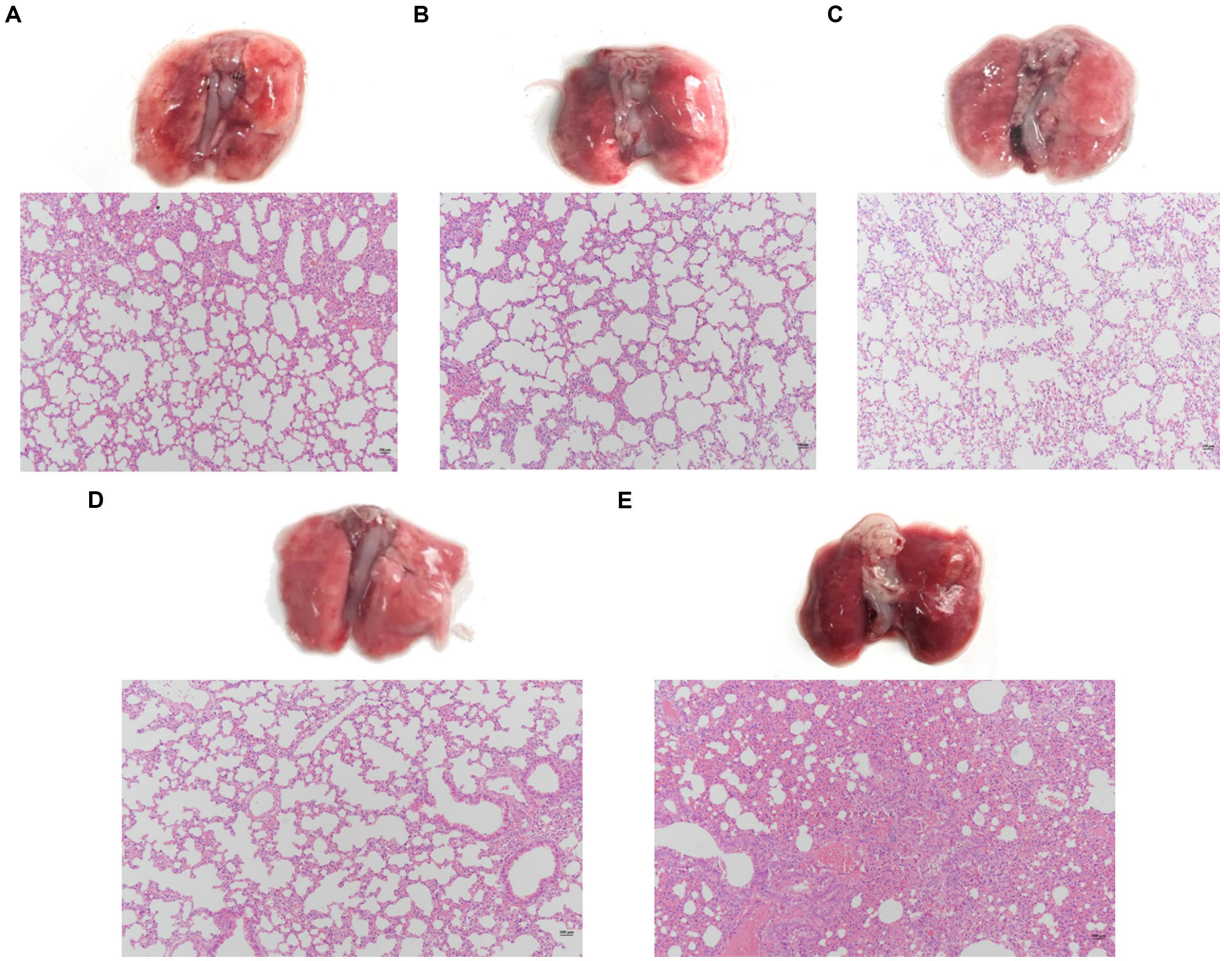
Pathological changes and histopathological sections of lung tissue. Two hours after infection, the lungs were removed from the mice that were, respectively, treated with phage YL1 **(A)**, phage YL2 **(B)**, phage cocktail **(C)** or PBS **(E)**. Healthy mice lungs **(D)** were used as control. Tissue samples were stained with HE.

Evaluate the effect of a single phage and a phage cocktail on the improvement of lung pathology in mice. On the surface, the lung tissue of normal mice appears pink, with a pale pink and smooth surface (Figure 7D). In contrast, the lungs of mice infected with PA27 appear dark red and reach the stage of red hepatoid characteristic of pneumonia (Figure 7E). In comparison, the lung tissue of mice treated with a single phage (YL1: Figure 7A, YL2: Figure 7B) in hemorrhagic pneumonia generally appears pink, with only certain areas showing dark red spots of hemorrhage. The lung tissue of mice treated with the phage cocktail (Figure 7C) in hemorrhagic pneumonia shows a significantly reduced presence of hemorrhage compared to the single phage treatment group. Based on histopathological analysis, normal lung tissue displays thin alveolar septa, with no infiltration of inflammatory cells in the alveolar spaces. In contrast, the lungs of mice with hemorrhagic pneumonia 24 h after infection with PA27 show severe inflammation, dilated capillaries, thickened alveolar septa, and congested alveolar walls, with almost no visible alveolar spaces. In comparison, the pathological changes in the lung tissue of mice treated with a single phage are significantly reduced, and the mice in the phage cocktail protection group exhibit the mildest lung pathology, indicating the most significant protective effect.

## 4 Discussion

Due to the inappropriate use of antibiotics and the emergence of multidrug-resistant bacteria, the treatment of bacterial infections has become increasingly complex and a global public health issue (Bachta et al., 2020). Currently, phages can accurately kill host bacteria without being limited by bacterial resistance, making them an ideal method for eliminating antibiotic-resistant pathogens and pathogenic bacteria in farmed production. In this study, two phages were isolated from mixed sewage samples from a farm in Shaanxi Province. Both phages have dsDNA genomes and are capable of lysing *P. aeruginosa* strains isolated from forest musk deer, producing 2 mm in diameter plaques. Based on transmission electron microscopy imaging, they were identified as lytic phages belonging to the *Podoviridae* family. However, compared to the 63.6% (21/33) lysis rate of phage Lx18, the two phages isolated in this study showed a narrower host range (Yin et al., 2022). In addition, phage ASP23 exhibited a lysis rate of 68% against clinically isolated strains of *P. aeruginosa* (Cui et al., 2023). When phages infect bacteria, the main structures involved in binding to the host bacterial surface receptors are the phage receptor binding proteins. Therefore, the host range of phages mainly depends on the diversity of their receptor binding proteins. To understand the interaction and infection mechanisms between phages and host bacteria, studying the RBPs of phages is necessary., In short, studying and modifying the structure of phage receptor binding proteins provides meaningful insights for further expanding the lysis range of phages. Due to the short latent periods and the absence of lysogenesis-related genes and bacterial-associated genes, the two phages meet the prerequisites for phage therapy. Additionally, both phages exhibit good resistance to temperature and acidic/alkaline environments, which is advantageous for their storage and utilization. Furthermore, the therapeutic study demonstrated that a single phage can control *P. aeruginosa* infection in mice, and a phage cocktail composed of the two phages has an even more significant therapeutic effect on mice with hemorrhagic pneumonia. Research has shown that a single intranasal administration of 10^8^ PFU/mL phage YH30 can provide 100% protection to minks against hemorrhagic pneumonia two hours after *P. aeruginosa* infection (Gu et al., 2016), and we also optimized our experiments based on these results. Since the host bacterium was isolated from the musk deer and used to establish a therapeutic model for hemorrhagic pneumonia in mice, this offers a better treatment option for antibiotic-resistant bacterial infections in wild and economically important animals.

At present, the rapid increase in antibiotic resistance among *P. aeruginosa* highlights the importance of developing new drugs for the treatment of bacterial infections. Holins and endolysins encoded by phages have the potential to serve as alternative agents to antibiotics. Currently, due to the presence of an outer membrane of *P. aeruginosa*, many exogenous endolysins cannot penetrate the peptidoglycan layer (Fischetti, 2010). However, the stability of the outer membrane can be disrupted through various methods, such as EDTA, malic acid, and other outer membrane permeabilizers (Briers et al., 2008). Studies have shown that when the outer membrane structure of bacteria is compromised, endolysins can kill gram-negative bacteria. Briers et al. (2011) reported that the combination of endolysin EL188 and EDTA reduced the CFU of *P. aeruginosa* in a short period of time. From an analysis of the safety of gut microbiota, phage endolysins can selectively kill target pathogens without damaging the coexisting bacterial communities (Nelson et al., 2012). Holins are present in most bacteriophages and are encoded by internal lytic genes. The holin protein has at least one transmembrane α-helical domain containing a highly charged hydrophilic C-terminal domain. During the late stage of infection, when the holin protein encoded by bacteriophage accumulates to a certain concentration on the cytoplasmic membrane, micropores are formed, and endolysin is released from the cytoplasm to reach the peptidoglycan of the bacterial cell wall. Based on the above perforation mechanism, holins themselves have the potential to be used as an adjunct therapy. The HolSMP protein from phage SMP has been reported to exhibit effective bactericidal activity against *Staphylococcus aureus* and *Bacillus subtilis* (Shi et al., 2012). However, in this study, further experiments are needed to determine the specific antibacterial effects of holins, which have 3 transmembrane domains, and soluble endolysins.

Bacterial resistance is continuously increasing, and multidrug-resistant infections have posed serious threats to the economic animal industry. Therefore, actively seeking phage therapy as an alternative to antibiotic treatment in cases involving multidrug-resistant bacteria is a promising direction. In this study, a mouse model of hemorrhagic pneumonia infection caused by multidrug-resistant PA27 was established to compare the protective effects of single phage or phage cocktail against drug-resistant lung infections. Of note, phage cocktail therapy can significantly reduce bacterial lung infections to a greater extent, which is consistent with the *in vitro* bacteriostatic ability of phages. In clinical studies of phage therapy, it is not possible to predict the strains that cause infections. Therefore, phage cocktails can provide a more comprehensive approach to prevent mixed infections with other pathogenic bacteria, thereby achieving the desired therapeutic effect (Gu et al., 2012). The combined use of phages and antibiotics can reduce the emergence of resistant and drug-resistant bacteria and is also an effective choice for preventing bacterial infections (Verma et al., 2010). It has been demonstrated that phage cocktail therapy can enhance efficacy, as it can lyse 86.7% of clinical isolates and exhibit synergistic effects when used in combination with antibiotics (Malik et al., 2021). Histopathological results showed that the phage cocktail treatment group had the mildest lung lesions, similar to the negative control group. This study demonstrates the potential of phage therapy for treating drug-resistant infections. In addition, the cure effect of the phage cocktail treatment group was better than that of the other two single-phage treatment groups, possibly due to the rapid targeting of the cocktail phage to eliminate the infection caused by the bacteria (Hooton et al., 2011).

## 5 Conclusion

In general, the phages YL1 and YL2, discovered in this study, belong to the *Podoviridae* family and exhibit good tolerance to low temperatures, pH, and UV irradiation. Genome analysis revealed the absence of virulence genes and lysogenic genes, making them suitable for clinical therapy. Additionally, the administration of these phages via intranasal route did not result in mouse mortality. Using a mice model of multidrug-resistant *P. aeruginosa* lung infection, it was observed that phage cocktail therapy was superior to single therapy, effectively controlling hemorrhagic pneumonia in mice, which was consistent with the results of the *in vitro* bacteriostatic assay. Therefore, the phages YL1 and YL2 have the potential to treat hemorrhagic pneumonia infections caused by *P. aeruginosa* and can be considered as alternatives to antibiotics.

## Data availability statement

The original contributions presented in the study are included in the article/Supplementary material. Further inquiries can be directed to the corresponding authors.

## Ethics statement

The animal studies were approved by the Executive Committee of Laboratory Animal Management and Ethical Review, Northwest A&F University. The studies were conducted in accordance with the local legislation and institutional requirements. Written informed consent was obtained from the owners for the participation of their animals in this study.

## Author contributions

YaZ: Writing – review & editing, Writing – original draft. RW: Writing – review & editing, Supervision, Data curation. QH: Writing – review & editing, Supervision, Data curation. NL: Writing – review & editing, Supervision, Data curation. LZ: Writing – review & editing, Software. ZY: Writing – review & editing, Supervision, Methodology. YeZ: Writing – review & editing, Supervision, Formal analysis, Conceptualization. XW: Writing – review & editing, Supervision, Resources, Methodology, Investigation, Funding acquisition, Data curation.
