# Supplementary figures and images for: Characterization of Pseudomonas aeruginosa bacteriophages and control hemorrhagic pneumonia on a mice model
Source: Front Microbiol. 2024 May 14;15:1396774. doi: 10.3389/fmicb.2024.1396774 (PMC11132263; doi:10.3389/fmicb.2024.1396774)

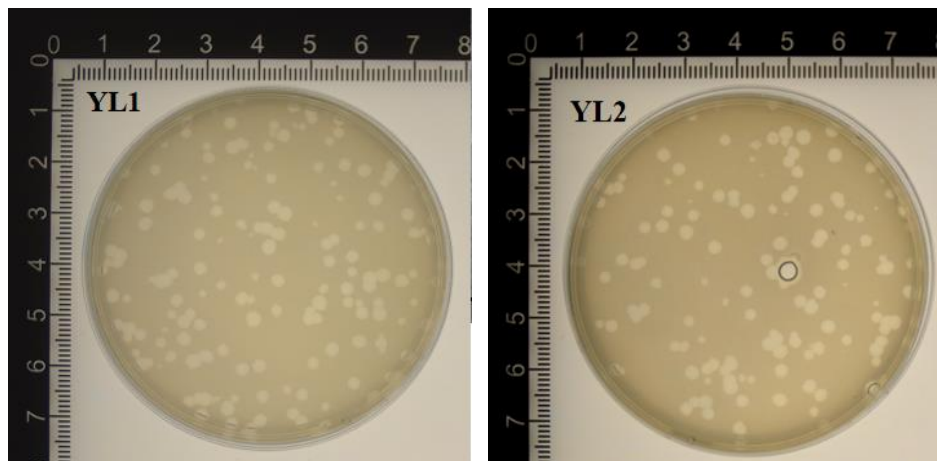

Supplementary Figure 1 *P.aeruginosa* bacteriophage YL1 and YL2 plaque

Supplement: Supplementary file 1 [file Data_Sheet_1.pdf]
