# Supplementary material for: Characterization of Pseudomonas aeruginosa bacteriophages and control hemorrhagic pneumonia on a mice model
Source: Front Microbiol. 2024 May 14;15:1396774. doi: 10.3389/fmicb.2024.1396774 (PMC11132263; doi:10.3389/fmicb.2024.1396774)
Supplement: Supplementary file 2 [file Data_Sheet_2.pdf]

Supplementary Table 1 Determination of cracking spectra by vB_PaeP_YL1 and vB_PaeP_YL2

Pseudomonas aeruginosa strains	vB_PaeP_YL1	vB_PaeP_YL2
Pseudomonas aeruginosa 27（Host）	+	+
PAf1	+	-
PAf10	-	+
PAf11	+	-
PA12	+	+
PAf13	+	-
PAf14	+	-
PAf15	+	-
PAf2	-	-
PAf3	-	+
PAf4	-	+
PAf5	-	-
PAf6	+	-
PAf7	+	-
PAf8	-	-
PAf9	-	-
PAO1	-	-
PAO10	+	+
PAO100	-	-
PAO101	+	-
PAO102	-	-
PAO103	+	+
PAO104	-	-
PAO105	+	+
PAO106	-	+

PAO107	-	-
PAO108	-	-
PAO109	-	-
PAO11	+	+
PAO110	+	+
PAO111	-	-
PAO112	+	+
PAO113	-	-
PAO114	-	-
PAO115	-	-
PAO116	+	-
PAO117	-	-
PAO118	-	-
PAO119	-	-
PAO12	+	+
PAO120	-	-
PAO121	-	-
PAO122	+	-
PAO123	-	-
PAO124	-	-
PAO125	-	-
PAO126	-	-
PAO127	-	-
PAO128	+	+
PAO129	-	-
PAO13	-	-
PAO130	-	-
PAO131	-	-
PAO132	-	-

PAO133	+	-
PAO134	-	-
PAO135	-	-
PAO136	-	-
PAO137	-	-
PAO138	-	-
PAO139	-	-
PAO14	-	-
PAO140	-	-
PAO141	-	-
PAO142	-	-
PAO143	-	-
PAO144	+	-
PAO145	-	-
PAO146	-	-
PAO147	-	-
PAO148	-	-
PAO149	-	-
PAO15	-	+
PAO16	-	+
PAO17	-	+
PAO18	-	-
PAO19	-	-
PAO2	-	-
PAO20	-	-
PAO21	-	-
PAO22	+	+
PAO23	+	-
PAO24	+	+

PAO25	+	+
PAO26	+	+
PAO27	-	-
PAO28	+	+
PAO29	-	-
PAO3	-	-
PAO30	+	+
PAO31	+	-
PAO32	-	-
PAO33	-	-
PAO34	-	-
PAO35	-	-
PAO36	-	-
PAO37	+	-
PAO38	+	-
PAO39	-	-
PAO4	-	-
PAO40	+	+
PAO41	+	-
PAO42	+	+
PAO43	+	-
PAO44	+	+
PAO45	-	+
PAO46	+	+
PAO47	-	-
PAO48	+	+
PAO49	-	-
PAO5	-	-
PAO50	+	+

frontiers

PAO51	-	-
PAO52	-	-
PAO53	-	-
PAO54	+	+
PAO55	+	+
PAO56	-	-
PAO57	+	+
PAO58	+	+
PAO59	-	-
PAO6	-	-
PAO60	-	+
PAO61	-	-
PAO62	+	+
PAO63	+	+
PAO64	+	+
PAO65	+	+
PAO66	+	-
PAO67	+	+
PAO68	-	+
PAO69	+	+
PAO7	+	+
PAO70	+	+
PAO71	+	+
PAO72	-	-
PAO73	+	+
PAO74	+	+
PAO75	-	-
PAO76	-	-
PAO77	-	-

PAO78	-	-
PAO79	+	+
PAO8	-	-
PAO80	+	+
PAO81	-	-
PAO82	+	-
PAO83	+	+
PAO84	+	-
PAO85	+	+
PAO86	+	-
PAO87	-	-
PAO88	-	-
PAO89	-	+
PAO9	-	-
PAO90	+	+
PAO91	-	-
PAO92	-	-
PAO93	-	-
PAO94	-	-
PAO95	+	-
PAO96	-	-
PAO97	-	-
PAO98	+	-
PAO99	+	+
Escherichia coli 2	-	-
Escherichia coli 201128	-	-
Salmonella 1	-	-
Salmonella 2	-	-
Staphylococcus aureus LS1	-	-

Note: "+" indicates that the host bacteria can be lysed by phage; "-" indicates that the host bacteria cannot be lysed by the bacteriophage
