# Supplementary material for: Characterization of Pseudomonas aeruginosa bacteriophages and control hemorrhagic pneumonia on a mice model
Source: Front Microbiol. 2024 May 14;15:1396774. doi: 10.3389/fmicb.2024.1396774 (PMC11132263; doi:10.3389/fmicb.2024.1396774)
Supplement: Supplementary file 3 [file Data_Sheet_3.pdf]

frontiers

Supplementary Table 2 Major functional proteins annotation of phage vB_PaeP_YL1

ORFs	Range	Start codon	Function[Best-match BLASTp Result]	Identities(%)	E-values	Accession
ORF6	4259_5548	ATG	tail fiber protein[vB_PaeP_MAG4]	96.97	0	YP_009290592
ORF10	7773_9941	ATG	DNA primase[Pseudomonas phage YH30]	99.72	0	YP_009226143
ORF11	9990_10724	ATG	Sak4-like ssDNA annealing protein[PA26]	95.90	2.00E-178	YP_009598413
ORF12	10751_11497	ATG	single strand DNA binding protein[LIT1]	98.39	7.00E-178	YP_003358459
ORF26	29439_30404	ATG	viron structural protein[YH6]	99.07	0	YP_009152578
ORF28	31131_32330	ATG	major head protein[PA26]	99.75	0	YP_009598429
ORF29	32365_33555	ATG	tail length tape measure protein[PAP02]	97.73	0	YP_010659112
ORF31	33963_36143	ATG	putative protal protein[LP14]	99.86	0	AWY02715
ORF32	36178_36597	TTG	deoxyuridine 5'-triposphate nucleotidohydrolase[MAG4]	99.28	1.00E-96	YP_009290618
ORF34	36933_37667	ATG	viron structural protein[Ab09]	100.00	0	YP_009031854
ORF35	37664_39316	ATG	terminase large subunit[vB_PaeP_TUMS_P121]	99.45	0	YP_010658980
ORF60	48340_49272	ATG	putative RNA polymerase[Pa2]	99.35	0	YP_009148199
ORF63	49860_51101	ATG	RNA polymerase large subunit[vB_Pae575P-3]	98.79	0	YP_010659168
ORF66	52274_52828	ATG	viron structural protein[vB_PaeP_TUMS_P121]	69.57	1.00E-85	YP_010659037
ORF74	55959_57029	ATG	ATPase[vB_PaeS_VL1]	98.60	0	YP_010658918
ORF77	58797_60035	GTG	putative DNA helicase[Pa2]	97.82	0	YP_009148216
ORF79	60562_63177	ATG	DNA polymerase[vB_PaeP_MAG4]	98.74	0	YP_009290574
ORF80	63174_63398	GTG	ferredoxin[Pa2]	100.00	8.00E-46	YP_009148219
ORF84	64398_64589	ATG	HNH endonuclease[YH6]	100.00	5.00E-40	YP_009152546
ORF85	64593_67121	ATG	RIIP lysis inhibitor[Pa2]	75.18	0	YP_009148224
ORF88	69267_69584	ATG	Holin[LIT1]	100.00	3.00E-69	YP_003358442
ORF91	70438_70950	ATG	Rz-like spanin[Pa2]	98.24	7.00E-117	YP_009148230
ORF92	70947_71471	ATG	lysozyme [vB_Pae575P-3]	95.40	7.00E-121	YP_010659198

Table 2 Major functional proteins annotation of phage vB_PaeP_YL2

ORFs	Range	Start codon	Function[Best-match BLASTp Result]	Identities(%)	E-values	Accession
ORF8	3845_5497	ATG	terminase large subunit[vB_PaeP_TUMS_P121]	99.45	0	YP_010658980
ORF9	5494_6228	ATG	viron structural protein[vB_PaeP_C2-10_Ab09]	100.00	0	YP_009031854
ORF11	6564_6983	TTG	dUTP nucleotido hydrolase[LP14]	99.28	9.00E-97	AWY02714
ORF12	7018_9198	ATG	putative protal protein[LP14]	99.86	0	AWY02715
ORF14	9606_10796	ATG	tail length tape measure protein[PAP02]	97.73	0	YP_010659112
ORF15	10831_12030	ATG	major head protein[PA26]	99.75	0	YP_009598429
ORF16	12088_12753	ATG	N4 gp55-like protein[LIT1]	99.55	2.00E-160	YP_003358473
ORF17	12757_13722	ATG	viron structural protein[YH6]	99.07	0	YP_009152578
ORF29	30855_31298	ATG	N4 gp48-like protein[LIT1]	97.96	3.00E-102	TP_003358461
ORF30	31295_31660	ATG	hypothetical protein BIZ95_gp65[PEV2]	100.00	2.00E-83	YP_009290599
ORF31	31664_32410	ATG	putative single strand DNA binding protein[Pseudomonas aeruginosa]	98.79	9.00E-179	SBT96838
ORF32	32437_33171	ATG	Sak4-like ssDNA annealing protein[PA26]	95.90	2.00E-178	YP_009598413
ORF33	33220_35388	ATG	DNA primase[YH30]	99.72	0	YP_009226143
ORF37	37613_38902	ATG	tail fiber protein[vB_PaeP_MAG4]	96.97	0	YP_009290592
ORF43	43750_44274	ATG	lysozyme[vB_PaeP_C2-10_Ab09]	99.43	5.00E-125	YP_009031822
ORF47	45637_45954	ATG	Holin[LIT1]	100.00	3.00E-69	YP_003358442
ORF51	50632_50823	ATG	HNH endonuclease[YH6]	100.00	5.00E-40	YP_009152546
ORF54	51393_51863	ATG	cytidine and deoxycytidylate deaminase zinc-binding region[vB_PaeP_MAG4]	99.36	1.00E-111	YP_00929056
ORF55	51823_52047	GTG	ferredoxin[Pa2]	100.00	8.00E-46	YP_009148219
ORF56	52044_54659	ATG	DNA polymerase[vB_PaeP_MAG4]	98.74	0	YP_009290574
ORF58	55186_56424	GTG	putative DNA helicase	97.82	0	YP_009148216
ORF61	58192_59262	ATG	ATPase[vB_PaeS_VL1]	98.60	0	YP_010658918

ORF69	62393_62947	ATG	viron structural protein[vB_PaeP_TUMS_P121]	69.57	1.00E-85	YP_010659037
ORF72	64120_65361	ATG	RNA polymerase large subunit[vB_Pae575P-3]	98.79	0	YP_010659168
ORF77	67274_67714	ATG	phosphatase[vB_PaeP_FBPa1]	100.00	2.00E-105	UVN14388
