# Supplementary material for: Characterization of Pseudomonas aeruginosa bacteriophages and control hemorrhagic pneumonia on a mice model
Source: Front Microbiol. 2024 May 14;15:1396774. doi: 10.3389/fmicb.2024.1396774 (PMC11132263; doi:10.3389/fmicb.2024.1396774)
Supplement: Supplementary file 4 [file Data_Sheet_4.pdf]

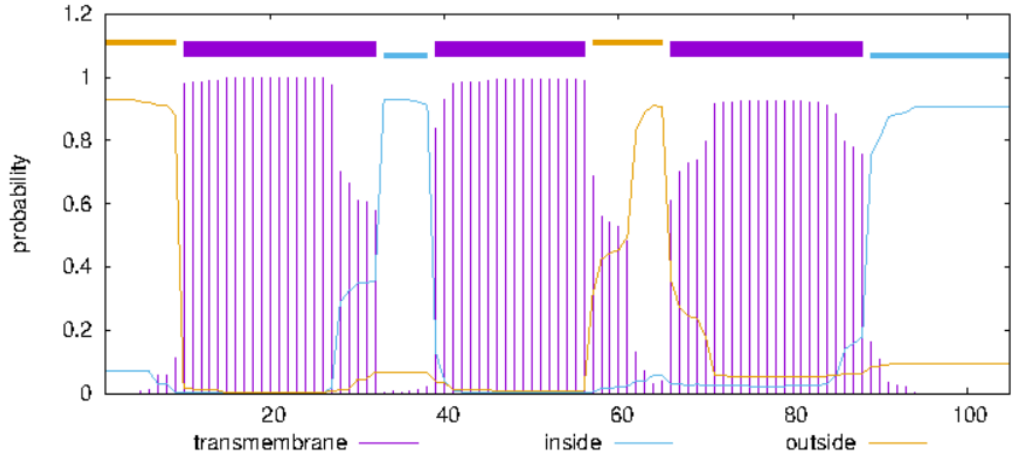

Supplementary Figure 2

Prediction of TMDs in HolYL1 by TMHMM

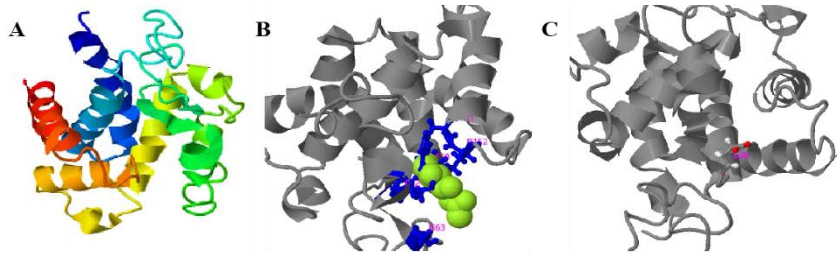

Supplementary Figure 3

LysYL1 tertiary structure prediction:A:3D model of LysYL1,B:Ligand binding sites of LysYL1,C:Active sites of LysYL1

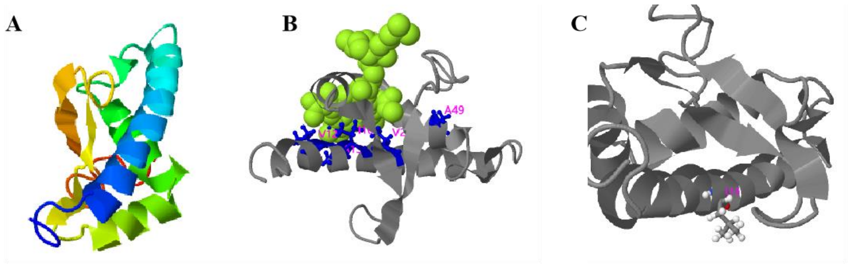

Supplementary Figure 4

HolYL1 tertiary structure prediction:A:3D model of HolYL1,B:Ligand binding sites of HolYL1,C:Active sites of HolYL1
